# Serum sodium improvement: change in Comprehensive Geriatric Assessment parameters in geriatric patients with hyponatremia

**DOI:** 10.1186/s12877-023-04299-x

**Published:** 2023-10-17

**Authors:** M. Kapoor, M. Pathania, M. Dhar

**Affiliations:** Department of Internal Medicine, All India Institute of Medical Sciences (AIIMS), Rishikesh, 249203 India

**Keywords:** CGA, Geriatric population, Hyponatremia, Comprehensive Geriatric Assessment

## Abstract

**Background:**

Hyponatremia presents with symptoms considered age-associated in the elderly. We assess the change in Comprehensive Geriatric Assessment (CGA) parameters after hyponatremia improvement in hospitalized geriatric patients.

**Methods:**

We took 100 hyponatremic and same number of eunatremic geriatric patients (> 60 years) who were comorbidity, presenting-complaints, and age-matched. Four CGA parameters were utilized, the new Hindi Mental State Examination (HMSE), Barthel’s index of activities of daily living (ADL), Timed up and go Test (TUG), and handgrip strength by hand dynamometer (HG). We analyzed these at admission and discharge, and their relationship with change in sodium levels.

**Results:**

Average age was 68.1 ± 5.8 years, with males constituting 75%. The CGA parameters demonstrated worse values amongst the hyponatremia than the normonatremia group. Severe hyponatremia group showed worse CGA scores in comparison with moderate and mild. With improvement in sodium level, the improvements in ADL, TUG, and HMSE scores were greater in the hyponatremia group (8.8 ± 10.1, 2.2 ± 2.5, and 1.7 ± 2.3 respectively) in comparison to the normonatremia reference group (4.7 ± 9.0, 1 ± 2.0, and 0.7 ± 1.3 respectively, P < 0.05).

**Conclusion:**

Our study is the first utilizing HMSE to assess change in cognitive ability with improvement in serum sodium levels in the Indian elderly. Hyponatremic patients show worse baseline CGA parameters, and hyponatremia severity correlates with worse motor and cognitive function. Improvement in the serum sodium level improves the CGA parameters. Correction of hyponatremia in the geriatric age group significantly impacts life quality.

**Supplementary Information:**

The online version contains supplementary material available at 10.1186/s12877-023-04299-x.

## Background

Hyponatremia is said to exist when the serum sodium value is < 135 meq/L [1]. It is the commonest electrolyte disturbance, seen in about 16–30% of in-hospital subjects [2]. Sodium has many functions, including maintaining blood volume, fluid homeostasis, membrane potentials, and acid-base balance [3]. Symptoms may include seizures, altered mental status, and drowsiness. Hyponatremia is seen in approximately 22% of the geriatric population in comparison with about 6% in others. Hyponatremia can present with subtle clinical symptoms, considered age-associated. This electrolyte abnormality may present numerous outcomes in the elderly, like common falling and balance disorders [4]. Other disorders associated include osteoporosis, falls, fractures, delirium, loss of cognition, and forgetfulness [5–7]. This may result in a reduction in living level. Still, as the geriatric age group has many underlying diseases like adrenal insufficiency, heart failure, liver disorders, and cancer, which can alter the cognition themselves, the impact of hyponatremia in cognitive changes remains unknown. Given this, we used four easy and routinely utilized comprehensive geriatric assessment (CGA) parameters for our study. These include Hindi mental state examination (HMSE), Barthel’s index of activities of daily living (ADL), Timed up and go Test (TUG test), and handgrip strength utilizing hand dynamometer (HG) for analyzing the relation of CGA parameters in the elderly with the degree of hyponatremia, along with the analysis of the impact of improvement in serum sodium levels on various CGA parameters. “Geriatric syndrome” is often used to refer to common health conditions in older adults (> 60 years of age) that do not fit into defined organ-system based categories and often have multifactorial causes. It includes conditions like cognitive impairment, delirium, incontinence, malnutrition, falls, gait disturbances, sleep disorders, and dizziness. Geriatric assessment refers to the evaluation by the primary care clinician, geriatrician, or a more intensive multidisciplinary program, also known as CGA.

## Methods

**Fig. 1:**
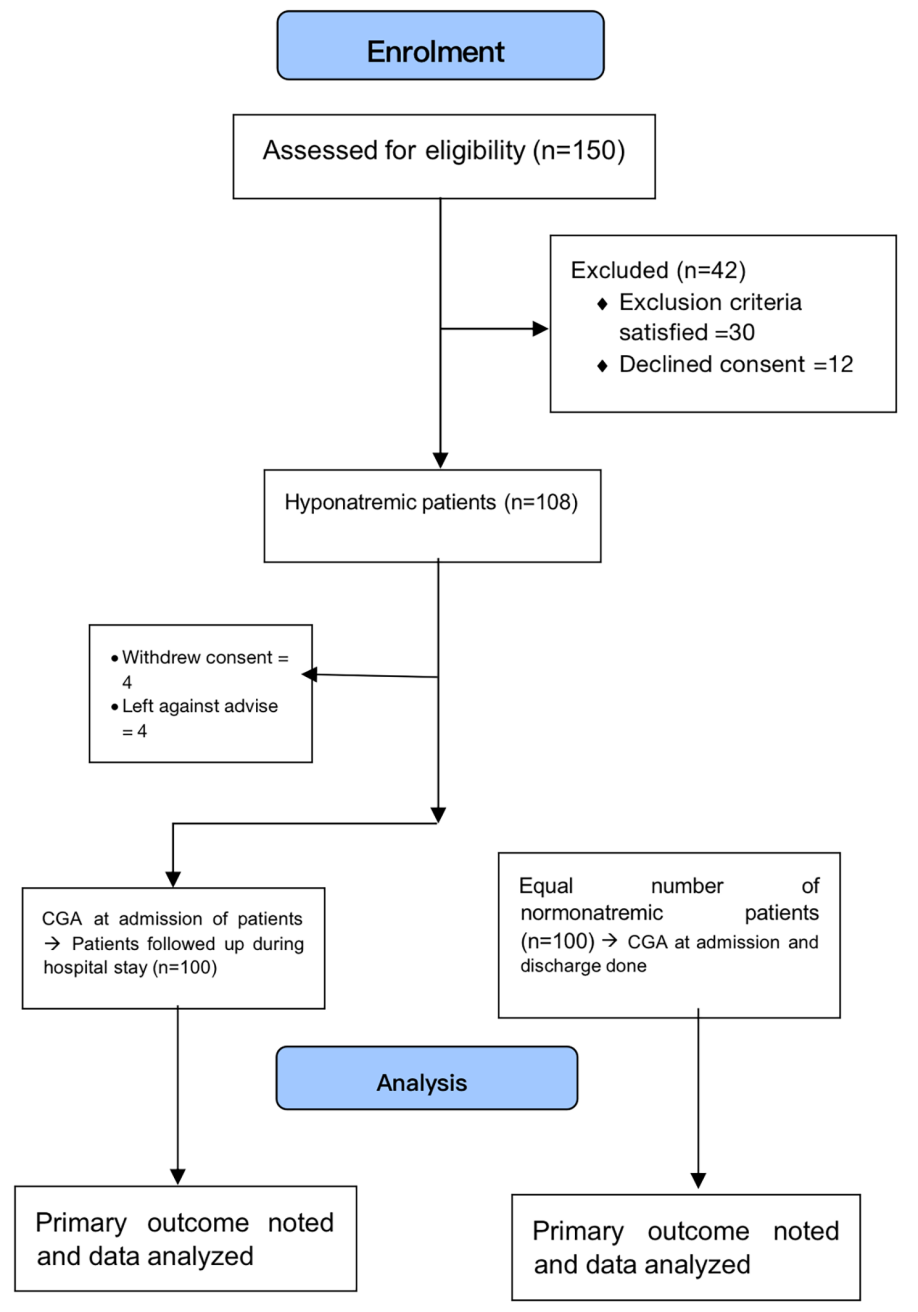
Study Flow

The study was conducted over one and half years, from April 2020 to October 2021, in a North India’s tertiary care center. The institutional ethical committee granted the clearance (No.- 379) and approved the protocol. All methods were carried out in accordance with local regulatory guidelines and regulations. Informed consent was obtained from all subjects and/or their legal guardian(s). As per our knowledge, no similar analysis has been done in the said territory. Because of the exploratory nature, sample size calculation was not done and we recruited 100 study subjects in our study. We assessed consecutive subjects ≥ 60 years from the geriatrics department of the institute. Their sodium level in the serum was < 135 meq/L. An equal number of normo-natremic persons matched for age, comorbidity, and reason of presentation to analyze the impact of serum sodium improvement per se in cognitive improvement compared to the improvement of the underlying diseases that may affect the brain function by themselves was taken. We excluded patients with other reasons of cognitive and motor impairments, including earlier cerebrovascular accidents, head injury, Parkinson’s disease, Alzheimer’s disease, meningoencephalitis, encephalopathy, muscle dystrophy, myelopathy, or in those patients where these parameters could not be assessed. All parameters were analyzed at admission and discharge, and their relation with hyponatremia severity was seen. (Fig. 1). The CGA domains were assessed by the residents, volunteers, and the nursing team after proper training for the same. In case of any discrepancy, the authors were communicated the same and resolution was ensured.

### Statistical analysis

Descriptive statistics are given for the categorical values in the form of frequencies. Comparison was done utilizing the Chi-square test. We used Student’s T-test and ANOVA test for comparing the numerical data. These analyses were depicted as mean ± standard deviation (SD). Analysis was done by Microsoft excel. A P-value of < 0.05 was taken as statistically significant.

### Study tools used

We assessed the cognitive abilities by utilizing the novel Hindi Mental State Examination (HMSE). Indo - U.S. Cross-National Epidemiology Study made an altered variant of the Mini Mental State Examination (MMSE), the HMSE. This is because the rural and the illiterate population of India speaks the Hindi language quite proficiently as compared to English. A score of 19 or below is taken as possible cognitive impairment on HMSE [8]. Barthel’s ADL and Hand Grip assessment by hand dynamometer were used for analyzing the motor function. The Barthel Index was introduced by Mahoney and Barthel in 1955 [9]. Scores below 20 mean “total” dependency, 21–60 suggest “severe” dependency, 61–90 “moderate” dependency, and > 90 “slight” dependency. Most groups utilize cut-off of 60/61. A hand dynamometer was utilized for handgrip detection [10]. For this, the participant sits upright on a chair with his/her feet supported. Typical values in elderly males and females were 29 and 16 kg, respectively [11]. Timed Up and Go (TUG) test was used to assess the mobility of the geriatric group. The subject gets up from the chair, walks three meters, turns, comes back, and sits in the chair. A score of more than 30 s means the subject is liable to fall. TUG value is usually < 12 s. [12].

## Results

We screened one hundred fifty geriatric hyponatremic patients (Sodium < 135 mmol/Litre), out of which 42 were excluded, four left hospital as per their demand, and four died within one day of admission. We took one hundred hyponatremic subjects and an equal number of eunatremic patients. On further analysis, it was seen that patients with severe hyponatremia had greater average age (71.60 ± 6.5 years) in comparison with moderate (66.60 ± 5.0 years) and mild (67.70 ± 5.5 years) hyponatremia (P < 0.05). Table 1 depicts the comparable basic variables of both groups except gender distribution, hemoglobin value, blood glucose value, and total leucocyte counts. As shown in Table 2, all CGA parameters were poor in the hyponatremia group, and the change in these parameters was statistically significant for all except handgrip. Table 3 shows the relation of various parameters according to the severity of hyponatremia. All CGA parameters were worse as the severity of hyponatremia increased.

**Table 1:** Characteristics of the study participants

Variables	Hyponatremia group	Normonatremia group	P-value
Males	74 (74%)	60 (60%)	0.03
Females	26 (26%)	40 (40%)	
Age (years)	68.1 ± 5.8	66.9 ± 5.9	0.15
**Comorbidities**			
T2DM	31 (31%)	25 (25%)	0.42
Hypertension	36 (36%)	31 (31%)	0.37
OAD	9 (9%)	12 (12%)	0.67
Chronic Kidney Disease	10 (10%)	6 (6%)	0.43
Other	14 (14%)	21 (21%)	0.25
**Diagnosis**			
CVS Disease	40 (40%)	41 (41%)	0.90
Upper respiratory tract infection	28 (28%)	37 (37%)	0.17
Urinary tract infection	8 (8%)	3 (3%)	0.13
Acute gastroenteritis	6 (6%)	6 (6%)	1
Anemia	5 (5%)	9 (9%)	0.27
DCLD	5 (5%)	4 (4%)	0.73
Others	12 (12%)	8 (8%)	0.35
Duration between assessment (Days)	13.7 ± 8.8	12.4 ± 8.2	0.3
Serum Potassium (meq/Litre)	4.6 ± 0.8	4.6 ± 0.5	0.7
RBS (mg/dL)	139.6 ± 57.8	121.1 ± 26.5	0.02
Hb (gm/dL)	10.7 ± 2.1	11.9 ± 2.4	0.01
TLC (per mm^3^)	11,734 ± 5706	8863 ± 4603	0.004
Na Test 1	129.7 ± 5.1	139 ± 3.4	2.4
Na Test 2	135.1 ± 2.8	140.2 ± 3.1	6.8
**∆ Na**	**5.5** ± 4.5	**1.3** ± 2.1	**4.6**

T2DM- Type 2 Diabetes Mellitus; OAD- Obstructive airway disease; CVS- Cardiovascular; DCLD- Decompensated Chronic Liver Disease; RBS- Random blood sugar; Hb- Hemoglobin; TLC- Total leucocyte count; Na- Sodium; ∆ Na- Change in sodium

**Table 2:** CGA values amongst the study participants and the change from admission to discharge

Variables	Hyponatremia group	Normonatremia group	P-value
ADL 1	71.6 ± 12.3	76.7 ± 11.5	0.001
ADL 2	80.2 ± 7.5	81.4 ± 6.5	0.18
**∆ ADL**	**8.8 ± 10.1**	**4.7 ± 9.0**	**0.002**
TUG 1	15.4 ± 3.4	15.4 ± 3.3	0.9
TUG 2	13.2 ± 2.8	14.4 ± 2.6	0.002
**∆ TUG**	**2.2 ± 2.5**	**1 ± 2.0**	**0.0002**
HG 1	9.2 ± 2.4	11.9 ± 3.6	1.6
HG 2	11 ± 2.2	12.3 ± 3.3	0.001
**∆ HG**	**1.8 ± 2.0**	**0.4 ± 1.6**	**4.5**
HMSE 1	23.4 ± 3.1	24.4 ± 2.4	0.01
HMSE 2	25.2 ± 1.7	25.1 ± 1.7	0.62
**∆ HMSE**	**1.7 ± 2.3**	**0.7 ± 1.3**	**0.00001**

HMSE: Hindi Mental State Examination; HG: handgrip; ADL: Barthel’s Activities of Daily Living; TUG test: Timed Up and Go Test; ∆-change

**Table 3:** Baseline parametres and CGA values in the patients in accordance to the severity of hyponatremia

Variables	Severity of hyponatremia			P-Value
	Mild (130 ≤ Na < 135)	Moderate (125 ≤ Na < 130)	Severe (Na < 125)	
Number	65(65%)	21(21%)	14(14%)	
Age	67.7 ± 5.5	66.6 ± 5.1	71.6 ± 6.6	0.04
Na Test 1	132.6 ± 1.1	127.5 ± 1.2	119.8 ± 5.5	9.2
Na Test 2	135.9 ± 1.5	134.6 ± 2.9	132.1 ± 4.1	1.7
**∆ Na**	**3.2 ± 1.6**	**7.1 ± 2.9**	**12.3 ± 6.6**	**1.05**
ADL 1	72.7 ± 10.5	70.4 ± 11.1	66.3 ± 18.4	0.18
ADL 2	79.6 ± 7.9	80.7 ± 6.4	81.3 ± 7.2	0.68
**∆ ADL**	**6.9 ± 7.8**	**10.2 ± 10.7**	**15.0 ± 14.2**	**0.01**
TUG 1	14.6 ± 3.5	16.4 ± 4.2	17.9 ± 3.4	0.003
TUG 2	12.9 ± 2.8	14.1 ± 3.1	13.1 ± 1.9	0.18
**∆ TuG**	**1.6 ± 1.5**	**2.2 ± 2.7**	**4.8 ± 4.1**	**0.0001**
HG 1	9.6 ± 2.2	8.6 ± 2.2	8.6 ± 2.9	0.14
HG 2	10.9 ± 2.1	10.4 ± 1.7	12.2 ± 2.3	0.03
**∆ HG**	**1.4 ± 1.5**	**1.7 ± 1.8**	**3.7 ± 3.4**	**0.0008**
HMSE 1	24.1 ± 2.5	22.6 ± 2.8	21.1 ± 4.0	0.0007
HMSE 2	25.4 ± 1.5	24.4 ± 1.7	24.9 ± 1.1	0.03
**∆ HMSE**	**1.4 ± 1.5**	**1.8 ± 2.3**	**3.8 ± 3.5**	**0.0007**

HMSE: Hindi Mental State Examination; ADL: Barthel’s Activities of Daily Living; TUG test: Timed Up and Go Test; ∆-change; Na: Sodium; HG: Handgrip

## Discussion

Hyponatremia is amongst the most familiar electrolyte disturbances seen in the population, especially in geriatric inpatients. Our study demonstrates the value of checking and improving serum sodium values, that too amongst the elderly age group. We found results of improvement in sodium values on the various CGA parameters. 14% of patients had severe hyponatremia (serum sodium < 125 meq/L) amongst the geriatric hyponatremic population in our study, moderate (125 ≤ Na < 130 meq/L) hyponatremia was seen in 21%, and mild hyponatremia (130 ≤ Na < 135 meq/L) was seen in 65%.

Persons with severe hyponatremia demonstrated greater average age (71.60 ± 6.6 years) in comparison with moderate (66.60 ± 5.1 years), and mild hyponatremia group (67.70 ± 5.5 years, P = 0.04). Similar findings were seen in other studies, meaning the prevalence of patients with severe hyponatremia becomes significantly greater as age advances. Factors contributing to the same include decline in renal function, increased water intake, reduced salt intake, and polypharmacy [13]. Change in serum sodium levels from admission to discharge was significantly greater in the severe group (12.3 ± 6.6 meq/L), as compared to the mild (3.2 ± 1.6 meq/L) and moderate groups (7.1 ± 2.9 meq/L). Notably, the improvement in ADL, TUG, and HMSE scores with serum sodium improvement was more in the hyponatremic group (8.8 ± 10.1, 2.2 ± 2.5, and 1.7 ± 2.3 respectively) when compared to the normonatremic reference group (4.7 ± 9.0, 1 ± 2.0, and 0.7 ± 1.3 respectively, P < 0.05). Although HG improvement was also more remarkable in the hyponatremic group, it was not statistically significant. The treatment measures (as both groups were matched for the primary diagnoses) and geriatric rehabilitation efforts were made identically in both the hyponatremic and normonatremic groups, hence these differences in CGA values are accounted for by the improvement of the serum sodium levels themselves.

Similar trends of more remarkable change in the ADL, TUG, HG, and HMSE values were seen in the various subgroups according to the severity of hyponatremia. All the severely hyponatremic patients demonstrated a greater change in the CGA parameters from admission to discharge after improving their serum sodium levels. This finding is very important, signifying that with the correction of sodium levels in the severely hyponatremic group, we can improve the motor and cognitive performance of geriatric patients quite significantly. The better performance in the discharge CGA parameters of the severe hyponatremia group may be explained by the subjective more enormous improvement from a comparatively poor initial performance.

Other studies like the INSIGHT study have revealed the result of sodium control (with Tolvaptan) on the results of various other tests evaluating neurocognitive domains in the elderly [14]. Renneboog et al. analyzed response times to various visual and auditory stimuli and revealed lower latency (by 8.6%) after correcting serum sodium levels [4]. Other studies have demonstrated that lower sodium levels associate with significant cognitive impairment, further cognitive decline, and the development of dementia [15, 16]. In our study, we also demonstrated significant motor performance improvement in the patients after improving serum sodium levels. Brinkkoetter et al. could not detect any reliable improvement in the motor power in the elderly [17]. This again demonstrated the clinical importance of our study. Falls in the elderly are a significant problem and one of the geriatric giants. The serum sodium level analysis must be done carefully in the geriatric age group presenting with falls, and sodium improvement may help the prevention of the same. This may reduce fractures, resulting in lower morbidity, mortality, and overall economic burden.

As per our knowledge, this is the first study of its kind studying the correlation of HMSE with hyponatremia. The baseline ADL, HG, and HMSE were lower in the severely hyponatremic group than the mild and moderate one, although it was statistically significant only for the HMSE value (24.1 ± 2.5 v/s 22.6 ± 2.8 v/s 21.1 ± 4.0; P = 0.0007). TUG test value was significantly higher in the severe (17.90 ± 3.40 s) when compared with the mild (14.60 ± 3.50 s) and moderate groups (16.40 ± 4.20 s, greater value meaning worse performance). Effect seen with ADL, TUG, and HMSE were not be reproduced with the other tests (HG here), implying that the exact impact of hyponatremia is yet incompletely understood. In spite of the proposed age-associated decrease in the renal capability for producing dilute urine, the expected lower reference level of serum sodium in the elderly was just lesser than the universal levels of 135 mEq/L [17]. This is an important finding, implying that even milder forms of hyponatremia should not be considered a normal variation with age. Our study has limitations. Patients with mental disorders like depression may perform sub-optimally in these CGA tests. Baseline CGA parameter results can be affected by other underlying diseases. However, we minimized this by including age, comorbidity, and primary diagnosis matched group which is normonatremic.

## Conclusions

In conclusion, the improvement in the serum sodium levels in the elderly group has positive effects on the neurological and cognitive functioning, and motor performance ability. We demonstrated statistically significant improvement in Barthel’s Activities of Daily Living index, an easily applicable test; Timed up and Go (TUG) test, a mobility measure ; and Hindi mental state examination (HMSE), a cognitive test specific for the Indian population. We also found that as hyponatremia becomes severe, it corresponds with poor motor and cognitive functions. Hence, serum sodium improvement holds a very important place in the elderly population given the impact of hyponatremia on the life quality. Further studies are required to analyze the mechanisms of hyponatremia’s impact on frail older people.

### Electronic supplementary material

Below is the link to the electronic supplementary material.

Supplementary Material 1

## Data Availability

The datasets used and/or analysed during the current study are available from the corresponding author on reasonable request.

## References

[1] Verbalis JG, Goldsmith SR, Greenberg A, Korzelius C, Schrier RW, Sterns RH, Thompson CJ. Diagnosis, evaluation, and treatment of hyponatremia: Expert panel recommendations. Am J Med. 2013;126:. 10.1016/j.amjmed.2013.07.006 10.1016/j.amjmed.2013.07.006 24074529

[2] Upadhyay A, Jaber BL, Madias NE. Incidence and prevalence of Hyponatremia. Am J Med. 2006;119. 10.1016/j.amjmed.2006.05.005 10.1016/j.amjmed.2006.05.005 16843082

[3] Overview of Sodium’s Role in the Body - Hormonal and Metabolic Disorders - MSD Manual Consumer Version. Accessed: June 29. (2021). https://www.msdmanuals.com/home/hormonal-and-metabolic-disorders/electrolyte-balance/overview-of-sodiums-role-in-the&#8230.

[4] Renneboog B, Musch W, Vandemergel X, Manto MU, Decaux G (2006). Mild chronic hyponatremia is associated with falls, unsteadiness, and attention deficits. Am J Med.

[5] Jamal SA, Arampatzis S, Harrison SL, Bucur RC, Ensrud K, Orwoll ES, Bauer DC (2015). Hyponatremia and fractures: findings from the MrOS study. J Bone Miner Res.

[6] Gosch M, Joosten-Gstrein B, Heppner HJ, Lechleitner M (2012). Hyponatremia in geriatric inhospital patients: Effects on results of a comprehensive geriatric assessment. Gerontology.

[7] Zieschang T, Wolf M, Vellappallil T, Uhlmann L, Oster P, Kopf D (2016). Assoziation von Hyponatriämie, Delirrisiko und Mortalität. Dtsch Arztebl Int.

[8] Tiwari SC, Tripathi RK, Kumar A (2009). Applicability of the mini-mental state examination (MMSE) and the Hindi Mental State Examination (HMSE) to the urban elderly in India: a pilot study. Int Psychogeriatr.

[9] Hartigan I (2007). A comparative review of the Katz ADL and the Barthel Index in assessing the activities of daily living of older people. Int J Older People Nursing.

[10] Gąsior JS, Pawłowski M, Williams CA, Dąbrowski MJ, Rameckers EA (2018). Assessment of maximal isometric hand grip strength in school-aged children. Open Med.

[11] Yoo J, Il, Choi H, Ha YC. (2017): Mean hand grip strength and cut-off value for sarcopenia in Korean adults using KNHANES VI. J Korean Med Sci. 2017, 32:868 – 72. 10.3346/jkms.2017.32.5.868 10.3346/jkms.2017.32.5.868 PMC5383622 28378563

[12] Clemens S, Gaunaurd I, Lucarevic J, Klute G, Kirk-Sanchez N, Bennett C, Gailey R (2018). Establishing the reliability and validity of the component Timed-Up-And-Go test to Determine Basic Prosthetic mobility in people with lower limb amputation. Can Prosthetics Orthot J.

[13] Imai N, Osako K, Kaneshiro N, Shibagaki Y. Seasonal prevalence of hyponatremia in the emergency department: impact of age. BMC Emerg Med. 2018;181–2018. 10.1186/S12873-018-0182-5 10.1186/s12873-018-0182-5 PMC6238288 30442112

[14] Verbalis JG, Ellison H, Hobart M, Krasa H, Ouyang J, Czerwiec FS et al. (2016) Tolvaptan and neurocognitive function in mild to moderate chronic hyponatremia: A Randomized Trial (INSIGHT). Am J Kidney Dis [Internet]. [cited 2021 May 27];67(6):893–901. Available from: https://pubmed.ncbi.nlm.nih.gov/26874645/10.1053/j.ajkd.2015.12.024 26874645

[15] Chung MC, Yu TM, Shu KH, Wu MJ, Chang CH, Muo CH, Chung CJ (2017). Hyponatremia and increased risk of dementia: a population-based retrospective cohort study. PLoS ONE.

[16] Nowak KL, Yaffe K, Orwoll ES (2018). Serum sodium and cognition in older community-dwelling men. Clin J Am Soc Nephrol.

[17] Brinkkoetter PT, Grundmann F, Ghassabeh PJ (2019). Impact of resolution of hyponatremia on neurocognitive and motor performance in geriatric patients. Sci Rep.

