# Supplementary material for: Serum sodium improvement: change in Comprehensive Geriatric Assessment parameters in geriatric patients with hyponatremia
Source: BMC Geriatr. 2023 Oct 17;23:666. doi: 10.1186/s12877-023-04299-x (PMC10580625; doi:10.1186/s12877-023-04299-x)

**Appendix**

Figure A: Hindi Mental State Examination (HMSE)


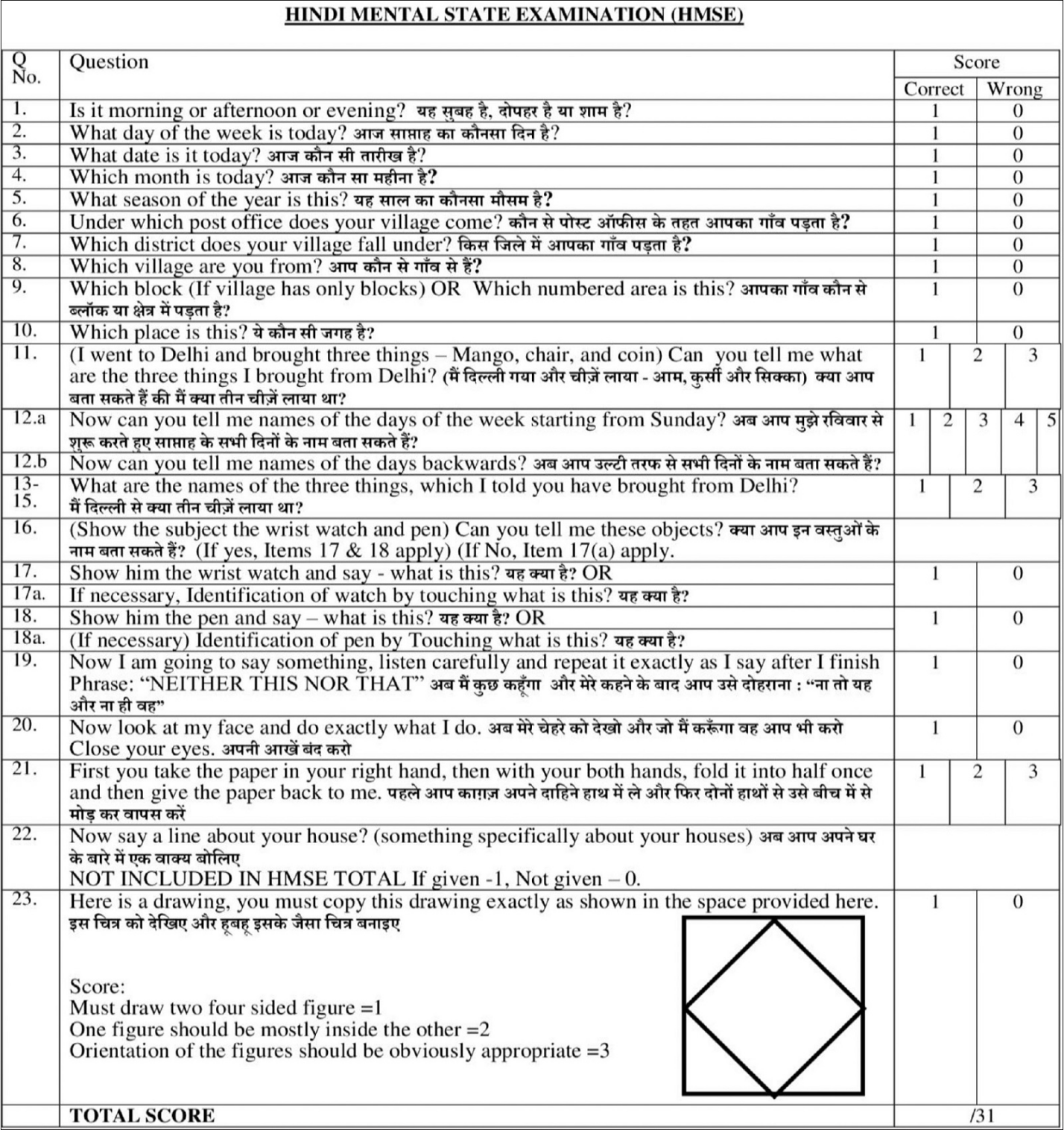


Figure B: Barthel’s index of activities of daily living


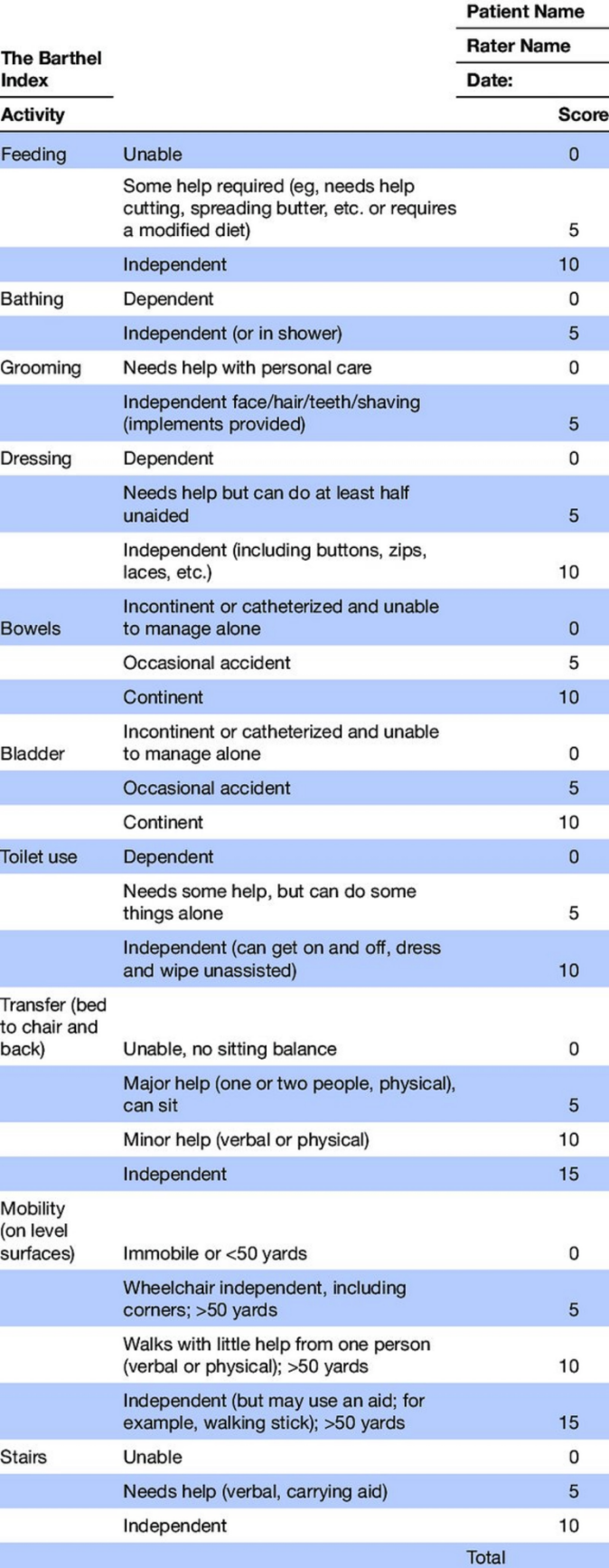


Figure C: Timed up and go test (TUG)


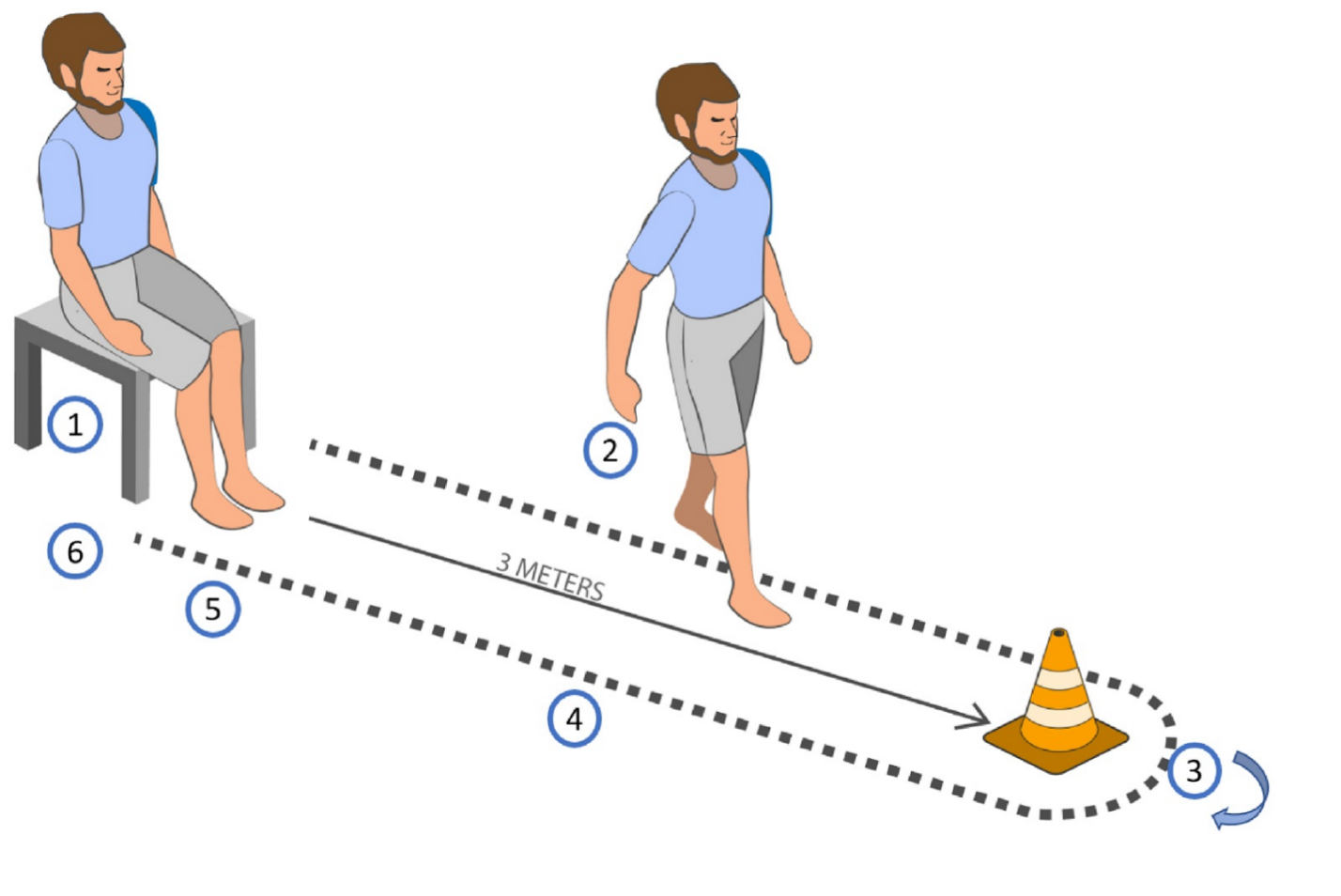


Figure D: Hand dynamometer


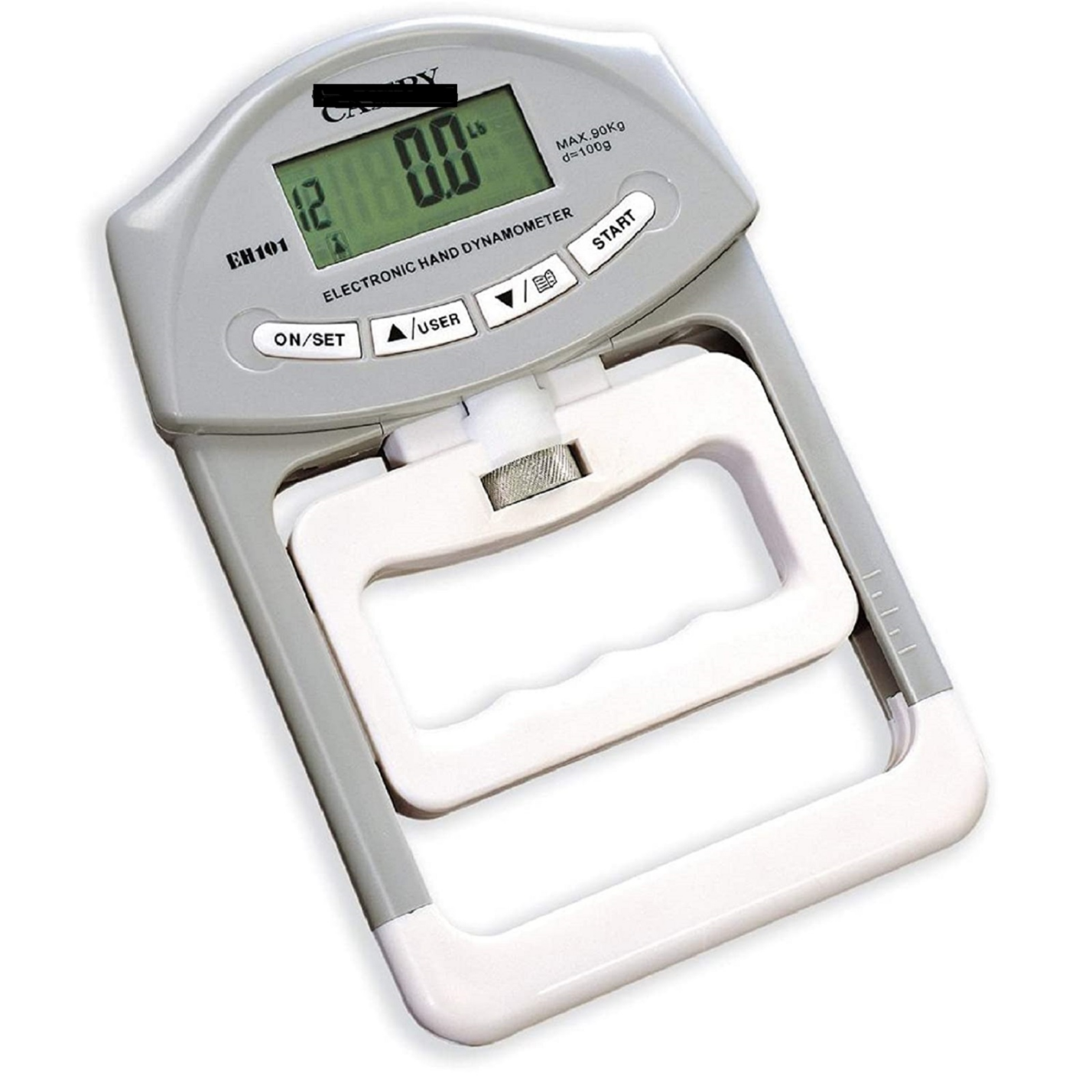

Supplement: Supplementary file 1 — Supplementary Material 1 [file 12877_2023_4299_MOESM1_ESM.docx]
